# Ayurvedic Management of Prurigo nodularis at scalp: A case report

**DOI:** 10.1016/j.jaim.2022.100668

**Published:** 2022-11-28

**Authors:** Danish Javed

**Affiliations:** AYUSH Department, AIIMS, Bhopal, MP, 462020, India

**Keywords:** Case report, Prurigo nodularis, *Alasaka*, Ayurveda

## Abstract

**Introduction:**

Prurigo nodularis (PN) is a chronic skin disorder of unknown origin which has the features of small nodules and papules along with intolerable itch leading to ulcers, bleeding and sensory neural loss in affected area. PN has quite resemblance with *Alasaka* in Ayurveda, which is a *vata-kapha pradhan kshudra kushtha*.

**Case summary:**

A 50 years old male patient suffering from PN was being treated on the line of Ayurveda just before the beginning of COVID pandemic. Due to sudden surge of first wave of corona virus pandemic and lockdown in India, the planed *virechana* (purgation) therapy was interrupted. We shifted our plan to mild type of *virechana* and conservative therapy after a short break of 10 days at patients’ home. However, the case was responded very well and significant clinical improvement was observed in Dynamic Pruritus Score (DPS), Numerical Rating Scale (NRS), Visual Analogue Scale (VAS), Verbal Rating Scale (VRS), Pruritus Severity Scale (PSS), Hamilton Depression Rating Scale (HDRS) and Dermatology Life Quality Index (DLQI).

## Highlights

• Prurigo nodularis a difficult to treat dermatological condition, has close resemblance to *Alasaka Kushtha* as per Ayurveda.
• This case was managed during lockdown situation of Covid 19 pandemic through Ayurveda line of treatment.
• Improvement in various subjective criteria like, DPS, NRS, VAS, VRS, PSS, HDRS and DLQI was noted significantly.

## 1 Introduction

Prurigo nodularis (PN), also known as the picker's nodule or chronic prurigo has firm excoriating dome like, oozing nodules present with severe itching and burning sensation in skin. It looks like insect bite or folliculitis. PN has some association with atopic dermatitis (AD) and environmental allergic substances [1]. Generally, this type of association is seen in young patients. But non-AD-PN is found in older age group and doesn't have sensitivity to environmental allergens. The patient feels piercing pain and severe itching. Due to excessive itching, these lesions often get converted into ulcers [2]. The lesions are generally in shape of dome like nodules. Often, they also have oozing and serous discharge. Usually, they are multiple and found in extremities. In some cases, sensory loss is often associated in form of neurotic stigmatization. Lesions remain continue even after the initial trauma in the form of itching. Management includes the use of anti-histaminics, intra-lesional triamcinolone and dressings with strong potency glucocorticoids. In complicated cases, thalidomide 50–100 mg may be useful. However, satisfactory treatment is still not available [3].

The symptoms and clinical features of PN are found quite similar to that of *kshudra kustha* (minor skin disorders) subtype “*alasaka*”, described in Ayurveda. In this case, main clinical features itching; nodular, reddish and oozing lesions were persisting on scalp and neck region. The predominant *doshas* (humours) found involved were *vata* and *kapha*, presenting their typical symptoms.

This case was planned for *sanshodhana chikitsa* (purification process) i.e. *virechana karma* (purgation therapy) on 24th march, 2020. *Snehapaan* (intake of medicated ghee) and *deepana-pachana* (appetite and digestion enhancing medication) was started six days prior. *Panchtikta ghrita* was administered to patient and *samyak snigdha lakshana* (symptoms of proper internal oleation) were also achieved. But due to outbreak of COVID-19, lockdown was announced in India and the purgation procedure could not be performed. However, patient was in our contact telephonically. We continued his *sanshaman chikitsa* (conservative management) on the Ayurveda line of treatment. He was managed somehow through tele-consultation. Finally, we achieved satisfactory results in this case.

The present case report, prepared as per the CARE guidelines demonstrates the management of a challenging case in pandemic time as all the routine OPD services were not available and our hospital was working as dedicated COVID Centre at that time.

## 2 Case presentation

### 2.1 Patient information

**Fig. 1:**
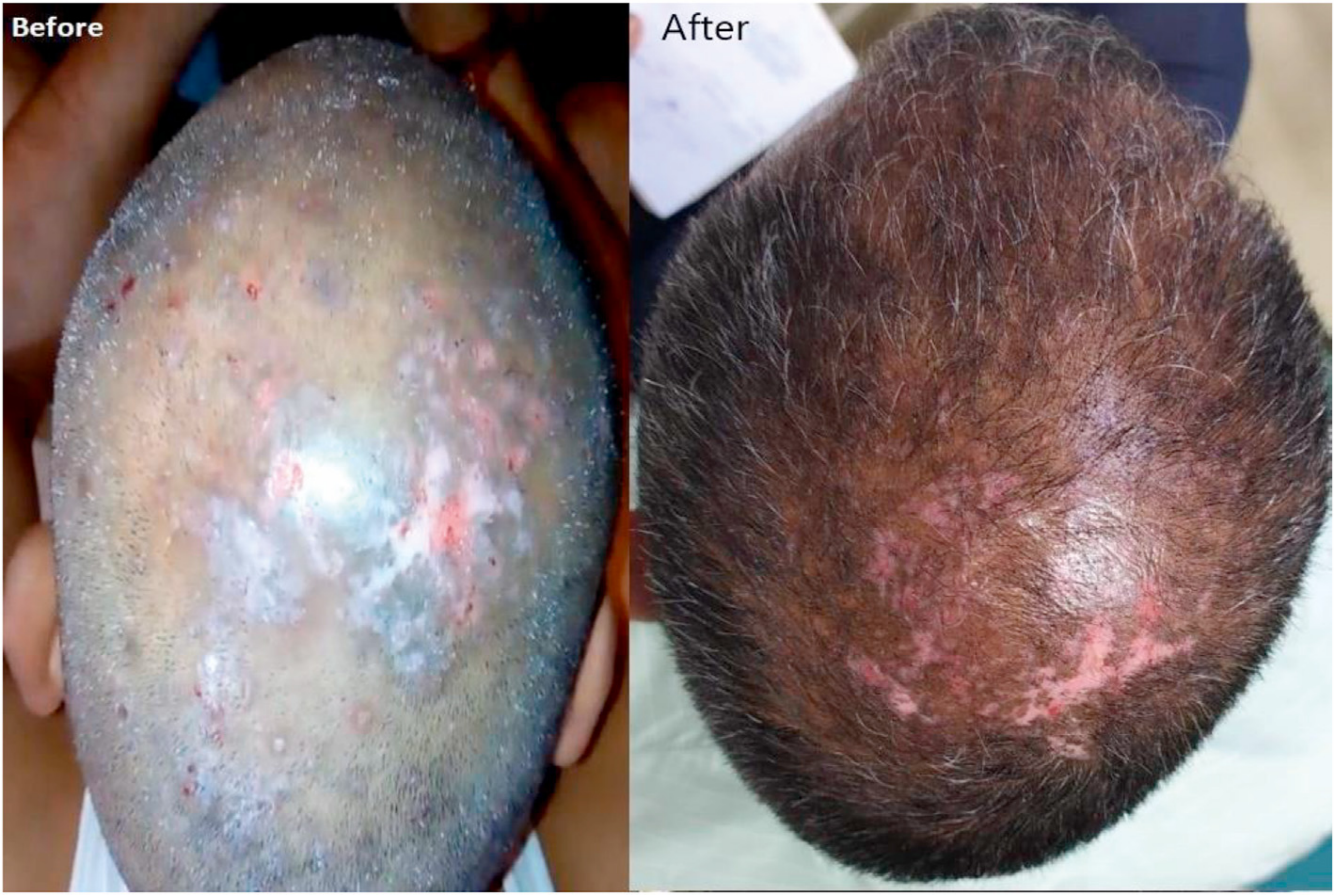
(a&b) Picture showing before & after clinical presentation of case.

A 50-year-old male patient was suffering with skin problems like tiny nodules on scalp with serous discharge and mild itching since last six years. It was mild symptomatic to begin with, but started producing intense itching over his scalp since last four years, later associated with intermittent localized pain and scanty purulent discharge from erosion spontaneously formed on the multiple tiny nodules since last one year along with numerous progressive itchy papules. He was treated with antihistamines, topical corticosteroids, and a short course of systemic steroids, but there was no improvement noticed by patient. Skin examination revealed multiple, hyper-pigmented papules and nodules from 1 to 3 mm size in diameter with some central erosion and crusts distributed on the scalp and nape of neck (shown in Fig. 1a). The hair, nails, mucous membranes, and lymph nodes were normal. Neurological examination revealed normal pin prick sensations, no muscle weakness, and no nerve enlargement. The patient had no past history of diabetes mellitus, hypertension, hypothyroidism, koch's, cadio-vascular disease, HIV, lymphoma, malignancy, psychiatric illness, depression, anxiety, atopic dermatitis, eczema or any other skin related or general disease. However, these lesions were having the tendency to aggravate in extreme summer or winter season and relieve in pleasant weather. Patient was fully conscious, well oriented, and normal vitals. He had no other complain of bowel movement, urinary difficulty, sleep, poor appetite, drug allergy, addiction, past surgery, trauma or any significant family history.

### 2.2 Present medical history

In January 2019, the patient visited first of all at allopathic dermatology OPD of AIIMS, Bhopal. Here, he was clinically diagnosed as a case of PN and modern allopathic treatment was started since then. However, skin biopsy was not taken, but other pathological tests were done to rule out other causes of disease. All the reports were found within normal limit (Hb 14.0 gm/dl, WBC 11.24 × 10^3^/μl, neutrophils 74%, lymphocytes 19%, monocytes 06%, eosinophil 1%, platelet 354 × 10^3^/μl, ECG normal sinus rhythm, X-ray chest normal, Anti-HCV Elisa, HbsAg Elisa negative, HIV-1 & 2 antibodies non-reactive, Blood urea 24 mg/dl, S. Creatinine 0.92 mg/dl, FBS 118.5 mg/dl, PPBS 114.8 mg/dl, ALT 22 u/l, AST 26 u/l, urine routine and microscopy- normal). These investigations helped to exclude other differential diagnosis like psoriasis, seborrheic dermatitis, tinea capitis, lichen simplex chronicus, recurrent folliculitis, lichenified plaques, scarring alopecia etc. Initially the course was started with antibiotics like cefadroxil, doxycycline; antifungal like itraconazole; antihistaminics levocetrizine and local application of ointments like clobetasol, beclometasone, ketoconazole, methoxsalen, aminobenzoic acid, fusidic acid, fluticasone etc. Steroids like prednisolone 20 mg and delazacort 6 mg was also given in due course of treatment. Thalidomide 100 mg dose was also prescribed twice a day to patient. But no any fruitful result was obtained in one year. In March, 2020, patient consulted in AYUSH department in Ayurveda OPD for the same complains.

### 2.3 Ayurvedic interpretation of the case

Patient was examined in OPD and detailed history was noted down as per the *vidhi vimarsha* (applied inference) of *Charaka samhita*. *Doshanusar parikshyabhava* (clinical picture) were examined to assess the predominance of *doshas* in this specific type of *kushtha* (skin disorder). Color of lesion was blackish, dusky or downy red *(vata)*, texture was oily or soft, white hairlines, elevated with smooth and fixed margins, copious, thick, white and slimy discharge *(kapha)*, macular and papular lesion *(kapha)*, rough and hard consistency *(vata)*, mild edema *(kapha)*, numbness present in few areas *(vata)*, cold to touch *(vata, kapha)*, pricking pain, loss of sensation *(vata),* Itching, burning sensation, feeling of burning, foul smell *(pitta),* Itching *(kapha),* Chronic onset with Slow progression *(kapha).* This clearly signifies that this lesion was predominantly *vata-kaphaj*. Presence of nodules with pruritus and redness is main clinical feature of *alasaka kshudra kushtha.* This case was found quite similar to *alasaka kshudra roga* as given by *Acharya Charaka* in *kushtha chiktsa adhyaya*.

### 2.4 Outcome measures

All symptoms present in this case was also evaluated on various subjective scores related to PN. Pruritus intensity was noted on Dynamic Pruritus Score (DPS)**,** Numerical Rating Scale (NRS), Visual Analogue Scale (VAS), Pruritus Severity Scale (PSS) and Verbal Rating Scale (VRS). Psychiatric co-morbidities were assessed through Hamilton Depression Rating Scale (HDRS). Dermatology Life Quality Index (DLQI) scale was used to observe Quality of life.

### 2.5 Interventions

The conservative treatment was started in OPD with *amritadi guggulu, maha manjishthadi kwath for oral intake; panchvalkal kwath and bactimo oil (containing maha marichyadi oil, maha trunak oil, neem oil, karanj oil, kapur oil, jaitun oil, tuvarak oil)* for local application for one week. *Panchkarma* therapy was planned and discussed with patient. It was advised him to start *panchkarma procedure* in next week. After taking informed consent, *panchtikta ghrita* (medicated ghee for internal oleation) in increasing dose of 10-20-40-80-160 ml oral once a day empty stomach, with *ushnodaka anupana* (luke warm water) was advised for five consecutive days. For the purpose of *deepana* and *pachana, chitrakadi vati* two tablets oral thrice a day with Luke warm water was also recommended. *Samyak snigdh lakshana* (symptoms of proper internal oleation) like *vatanulomya* (relieving flatulence), *diptoagni* (increased appetite)*, varch sasnigdham asamhata* (Softness and unctuousness in stool)*,* and *mardav snigdhata cha-ange* (softness and oiliness in body)*,* were observed after five days*. Snehana* and *sarwanga swedana* (Oil massage and steam bath) was given for next three consecutive days. *Virechana karma* was planned on next day, which could not be possible due to lockdown. In this situation, we contacted to patient telephonically and advised to take Luke warm *triphala kwath* 20 ml orally in morning time empty stomach for next three consecutive days. Three to four *vegas* of *virechana* were observed each day. *Sansarjana karma* diet (gradual dietary regime) was then followed for next 10 days telephonically again. During this period, we kept close observation on his appetite, digestion and bowel movement. Suggestions were provided up to the normal diet was achieved by patient. After which oral medicines were started to take as earlier. Finally, the desired result was achieved in terms of reduced itching, decreased secretions, and drying of nodules started just after the *virechana* therapy without any procedural complications. Patient was in our contact telephonically during this period and he could come for follow up on 9th Oct, 2020. He was feeling well and all the previous symptoms were relieved.

### 2.6 Time-line and follow-up

The case was further assessed on same clinical parameters and changes were observed in due course of time (See: Table 1, Table 2, Fig. 1b). Almost the lesions on scalp were healed completely. Itching, oozing and nodules were reduced significantly. He is still in our follow-up and no relapse is observed till date (See Table 3). Gradually the dosage of oral medicines has been also reduced.

**Table 1:** Subjective changes observed on different parameter.

Parameter	Scale	B.T.	A.T.	D.	%age change
Pruritus intensity	Dynamic Pruritus Score (DPS)	4	8	4	100
	Numerical Rating Scale	8	1	7	87.5
	Visual Analogue Scale	8	1	7	87.5
	Verbal Rating Scale	4	1	3	75
	Pruritus Severity Scale	16	5	11	68.7
Psychiatric co-morbidities	Hamilton Depression Rating Scale (HDRS)	6 (no depression)	6	0	0
Quality of life	Dermatology Life Quality Index (DLQI)	9	1	8	88.89

**Table 2:** Changes in objective assessment criteria.

S.No.	Symptoms	Before	After
1	Itching	Severe	Mild/No
2	Macular Nodules	Severe	Mild/No
3	Oozing	Mild	No
4	Bleeding	Mild	No

**Table 3:** Timeline of *Ayurvedic* management.

S.No.	Date	Medicine/Therapy	Dose/Remarks
1	6 Mar, 2020	• *Amritadi guggulu*	• 2 Tablets oral twice a day, with water, after meal
		• *Maha manjishthadi kwath*	• 15 ml oral, twice a day, with water, after meal
		• *Panchvalkal kwath*	• For *prakshalana* (head wash)
		• *Bactimo oil containing maha marichyadi oil, maha trunak oil, neem oil, karanj oil, kapur oil, jaitun oil, tuvarak oil,*	• Twice a day, for local application
2	15–19 Mar, 2020	• *Panchtikta ghrita,*	• 10-20-40-80-160 ml
		• *Chitrakadi vati*	• 2 tablets orally, thrice a day
3	20–23 Mar, 2020	*Snehana* + *sarwanga swedana*	*Mahamarichyadi tail + til taila*
4	24–26 Mar, 2020	*Mridu virechana* for 3 days	Could not be possible due to country wide lockdown. Only *triphala kwath* 20 ml was administered for consecutive 3 days. 3–4 *vegas* came daily.
5	27 Mar, 2020 onwards	*Sansarjana krama*	All treatment was stopped for 10 days. Patient was advised to take light diet.
6	10 Apr, 2020	*Sanshaman chikitsa* was started as above in point no 1.	Online consultation was given throughout the six-month period.
7	9 Oct, 2020	Follow-up visit	Gradually the symptoms subsided

### 2.7 Dietary regimens

It was instructed to patient to take light, non-spicy, less salty, low fat and fresh diet. He was advised to take fruits, salad, and liquids frequently. Fast food, beverages, non-vegetarian, heavy and fried foods were restricted. Use of chemical-based cosmetics products was also restricted in the form of soap, cream, oil, face wash or perfumes.

## 3 Patient's perspective on treatment received

Sir has successfully treated my scalp prurigo with Ayurvedic medications. Before his consultation, I had the complaints of severe itching and burning over my scalp region near the head and neck. For this problem; I initially consulted to allopathic doctors in dermatology OPD. They were prescribing me allopathic tablets, lotions, creams and injections also. I took all these medicines for about one year, but did not get any benefit. Then I decided to opt some other system of medicine. So, I turned toward Ayurveda and consulted in AYUSH OPD. Here, I was examined by Ayurveda doctor. Sir has prescribed me kadha, oil, and some other Ayurvedic medicines for my problem. Due to COVID-19 and lockdown, Sir has supported me telephonically and treated very well. I was under his treatment in last seven to eight months. Slowly my scalp was started healing and now, I am feeling much better. Really, I am much more thankful to Sir, Thanks & regards.

## 4 Discussion

PN is a chronic disease which has hyperkeratotic papules or nodules with severe itching. There is an irrepressible cycle of pruritus and scratching, which leads to excoriation, lichenification or crust formation on the affected part. Neuronal proliferation, raised local concentrations of neuropeptides, small-fiber neuropathy, presence of eosinophils and mast cells are considered as causative pathology leading to severe pruritus. Still, the patho-physiology behind dermal neuronal hyperplasia or neuronal plasticity in PN is not much clear [3]. However, it has been observed that there is a link between stressful situation like anxiety, depression and pruritus. The condition worsens due to which many times, when patients unknowingly make excoriations in lesions through excessive itching, which may also form large size ulcers. The dermatological conditions, in which there is a close relation of anxiety and pruritus, Cognitive behavioral therapies (CBT) may also be helpful to combat the situation. Yoga and meditation may also be suggested in such conditions as they may play a key role to break this cycle of anxiety associated with the dysfunctional thought [4].

Many herbal drugs are found useful in treatment of prurigo. *Cocculus hirsutus, Lawsonia inermis, Prunus amygdalus, Saussurea lappa***,** *Capsicum annum* etc. are known as effective cure in PN [4]. Active constituent in *C. annum* i.e., 0.025%–0.3% of capsaicin has been reported as therapeutic agent in PN, if we use 4–6 times daily [5]. One study suggests that treatment of *C. annum* extract for 22 weeks to 33 months may reduce the symptoms of PN [[4], [5]].

If we talk about *Ayurvedic* management, *virechana* has been said as a good therapeutic option in pure *pitta vikaras* as well as *kaphaj dushti* located in *pitta sthana*, as per the *Vagbhatta* [6]. *Acharya Bhela* has also mentioned that *virechana* acts in *sannipatik* condition also. If *virechana* is given in prurigo patients, it is reported that within three to four months, lesions may be reduced up to near normal. *Virechana karma* has also been found quite effective in treatment of chronic wounds [7]. However, many conservative treatments (*sanshaman chikitsa*) are also available for skin disorders as *Ayurvedic* formulations like *kaishor guggulu, amritadi guggulu, arogyavardhani vati, khadiraristha* etc [8].

In the present case, the situation was become challenging due to sudden lockdown and cessation of all type of health services during COVID-19 period. However, the *snehana* was properly given to patient and all *doshas* also became *utkleshit* after *abhyanga* and *swedana*. The lockdown was started just at the day when *virechana* was planned. We made a telephonic consultation in such situation and mild laxative was advised to take in ambulatory situation in the form of *triphala kwatha*. As *doshas* got exaggerated and it was must to compel out them from body to avoid any *upadrava*, it was decided to give *triphala kwath* on next three consecutive days to achieve desired result. After three days of mild *virechana karma, sansarjana karma* was also planned accordingly for next one week. After proper *agnideepana, sanshaman aushadhis* were started in previous doses. Patient could not come physically at hospital during this period, but virtual communication meetings make it possible to guide him properly and even proper support was also given to boost his moral.

After some time, when situations became near normal, patient came for follow up visit with complete recovery in his symptoms. The scalp lesion was subsided and healed up. The findings were noted down as per the previous parameters and a sharp decline was observed in all the subjective parameters.

Here, we used *amritadi guggulu* and *maha manjishthadi kwath* for oral conservative treatment. *Amritadi guggulu* is a polyherbal compound which mainly contains *giloy, triphala, trikatu, trivritta, danti, vidang and guggulu. Maha manjishthadi kwath* (decoction) is also a polyherbal preparation containing mainly *manjishtha, mustaka, karanjbeej, giloy, haridra, daruharidra, nimba twaka, sariva* etc. They may have worked as anti-inflammatory, immunomodulatory, anti-histaminic and anti-microbial. *Panchvalkal kwath* is also combination of five astringent herbs viz *nyagrodh, udumbar, ashwatha, parisha and plaksha.* They have *shothahar, vranaropan, kapha shamaka and rakta shodhaka* properties. Bactimo oil is an *Ayurvedic* proprietary medicine, which is used in dermatitis, itching, bacterial and fungal infections. Definitely, their use was highly supportive during course of treatment and as to prevent remission of the lesion later on. The beneficial aspect in this study was observed that other steroids, anti-allergic, anti-inflammatory, anti-biotic, antifungal drugs were fully withheld and not required at all.

*Sanshodhana karma* plays a very significant role in skin diseases, as *doshas* get *sthan-sanshraya* in between the *twak* and *mansa dhatu* with the help of *rakta* and *lasika*. Causative factors which aggravate this pathogenesis are mainly *atilavana, atiamla, virruddha ahar, guru snigdha anupana, atidravapaan, sneh atisevan, asatmya ahar, ajirn ahar, chilchim fish with milk, gramya-anup-audak mansa, ati madyapaan* etc [8]. The main paths of *doshas* expulsion are only two, either by means of *rakta-visravan* (bloodletting) or by *sanshodhana karma*. *Vaman* plays good role in *kaphaj* skin problems, while *pitta doshas* are removed gently by *virechana karma* in *pitta pradhana kushtha. Acharya Charaka* has also mentioned that in *vata-kaphaj kushtha*, the line of treatment of *pittaj kushtha* should be followed *(mārutakapha kuṣṭhaghnaṁ karmōktaṁ pitta kuṣṭhināṁ kāryam | Charaka kushtha chikitsa 58)* [9]. Here, that is why we also opted *virechana karma* in a case of *alasaka,* which is considered as *vata kaphaj kushtha.*

In the present case, it was observed that intensive one day *virechana* therapy may be replaced with three to four days mild laxative *virechana* therapy in some extra-ordinary situations too. However, this is a single case study and promising result was found, it may be planned in larger sample size to prove the efficacy of this variation. The lesson learnt in this case was that the *sanshodhana karma* in PN is highly promising and this deviation of routine practice may be established in order to achieve better compliances.

## 5 Conclusion

The case report demonstrates clinical improvement in PN with the help of *sanshodha karma* and *sanshaman aushadhi*. *Virechana* has shown excellent response in this case. Similar approach in a larger cohort may be useful to establish its efficacy in such chronic skin disorders.

## 6 Limitations

In the cases of PN, role of *Panchkarma therapies* like *snehapaana, swedana, virechana, rakta-mokshana* etc. cannot be ignored. Due to lockdown and COVID protocol in hospital, we could not administer *panchakarma* therapy in a proper manner in this case, however the *sanshaman chikitsa* along with minor modifications in therapy schedule was found quite satisfactory.

## Declaration of patient consent

The author certifies that he has obtained the appropriate patient consent form. In the form, the patient has given his consent for his images and other clinical information to be reported in the journal. The patient understands that name and initials will not be published and due efforts will be made to conceal identity, but anonymity cannot be guaranteed.

## Financial support and sponsorship

Nil.

## Conflict of interest

None.

## Author contributions

**DJ:** Conceptualization, Methodology, Investigation, Writing - Original Draft, Review & Editing.
